# Recent Advances in Particulate Matter and Nanoparticle Toxicology: A Review of the *In Vivo* and *In Vitro* Studies

**DOI:** 10.1155/2013/279371

**Published:** 2013-06-20

**Authors:** Abderrahim Nemmar, Jørn A. Holme, Irma Rosas, Per E. Schwarze, Ernesto Alfaro-Moreno

**Affiliations:** ^1^Department of Physiology, College of Medicine and Health Sciences, United Arab Emirates University, Al Ain 17666, UAE; ^2^Department of Air Pollution and Noise, Norwegian Institute of Public Health, N-0403 Oslo, Norway; ^3^Aerobiology Laboratory, Atmospheric Sciences Center, Universidad Nacional Autónoma de México, 04510 Mexico City, DF, Mexico; ^4^Environmental Toxicology Laboratory, Instituto Nacional de Cancerología, México. Avenido San Fernando 22, Tlalpan, 14080 Mexico City, DF, Mexico

## Abstract

Epidemiological and clinical studies have linked exposure to particulate matter (PM) to adverse health effects, which may be registered as increased mortality and morbidity from various cardiopulmonary diseases. Despite the evidence relating PM to health effects, the physiological, cellular, and molecular mechanisms causing such effects are still not fully characterized. Two main approaches are used to elucidate the mechanisms of toxicity. One is the use of *in vivo* experimental models, where various effects of PM on respiratory, cardiovascular, and nervous systems can be evaluated. To more closely examine the molecular and cellular mechanisms behind the different physiological effects, the use of various *in vitro* models has proven to be valuable. In the present review, we discuss the current advances on the toxicology of particulate matter and nanoparticles based on these techniques.

## 1. Introduction

Exposure to particulate matter (PM) is associated with increases in visits to emergency rooms and mortality [1]. The Meuse valley fog of 1930 [2], the Donora smog incident of 1948 [3], and the London great smog event of 1952 [4] triggered the studies of health effects related to the exposure to PM in large cities and later on the legislation regarding the level limits of PM. For instance, in the US, the Clean Air Act was enacted in 1972.

**Figure 1:**
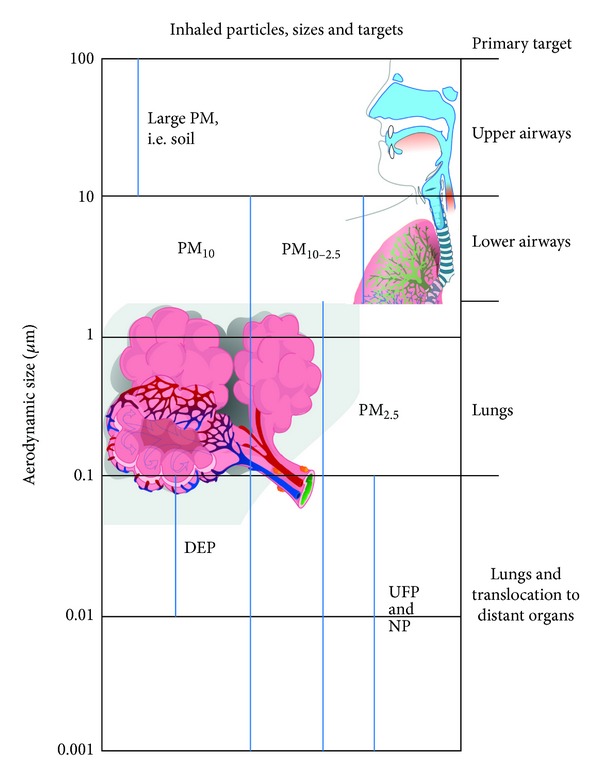
Schematization of the size and main target for particulate matter and nanoparticles.

Inhaled particles penetrate into the respiratory tract where they target different anatomical sites, depending among other properties on the aerodynamic size. Particles are categorized according to aerodynamic size, PM_10_, thoracic particles, (≤10 *μ*m) and PM_2.5_ (≤2.5 *μ*m), or fine fraction. The particles with a range of aerodynamic sizes between 10 and 2.5 *μ*m (PM_10-2.5_) are know as coarse fraction. If the aerodynamic size is equal or less than 0.1 *μ*m, the particles are called ultrafine particles (UFP), and one of the main sources of this type of primary particles is diesel exhaust (DEP). Engineered particles, measured by their geometric size and with at least one dimension smaller than 0.1 *μ*m, are known as nanoparticles (NP) [5]. The primary anatomical target of particles with different sizes is summarized on Figure 1.

Air Quality Standards have been adopted by many countries around the world to protect public health and welfare against the adverse effects of air pollution. In fact, member countries of the World Health Organization (WHO) have adopted a constitution that sets guidelines on air pollutants. The WHO, which has representation from nearly 200 countries, recommends daily PM_10_ concentrations not to exceed 50 *μ*g/m^3^ [6]. Many countries, however, have chosen to set Air Quality Standards that are more relaxed or more stringent than the WHO Standard. Air Quality Standards are generally created or revised according to national policy and scientific information that demonstrates a plausible association between health-related illnesses and exposure to pollutants. The limits for PM_10_ and PM_2.5_ that are used in different countries and regions are shown in Table 1 (Modified from [6]).

Despite all the efforts for measuring the health impact of inhaled particulate matter, we are still far from fully understanding all the effects and mechanisms related to those effects, and also, we still do not understand what is the role of the length (size), shape, and composition of particles in their biological effects. In the present paper, we reviewed the relevant information related to three main aspects of the problem: (1) the determination and role of the chemical and biological components of particles, (2) the evaluation of the *in vivo* effects, both at pulmonary and systemic targets, and (3) the evaluation of the mechanisms of the cellular effects of particles with different sizes, shapes, and composition. Among the large amount of articles that are published in these fields, we choose those that we consider are helping to understand the different problems and also those articles that are opening new questions, pushing the limits of our knowledge forward.

## 2. Characterization of Particles

Combustion particles from traditional fuels (biomass, coal, wood, crude oil, and diesel with high content in sulfur) are now found in much lower concentrations in air than 30–40 years ago due to improved and cleaner technology. The relative size distribution has changed, and other pollutants have gained prominence, such as ultrafine PM (UFP) [7]. These new and lighter airborne PM is found not only in big cities but also in large and small towns. Interestingly, they differ in composition with regard to various heavy metals and polycyclic aromatic hydrocarbons (PAHs) and are often found to have a higher oxidative and toxic potentials. 

Depending on the source and composition of the PM different subsets of components may be found on different fractions. PM_2.5_ comprises the soot fraction and particles grown from the gas phase with subsequent agglomeration. PM_2.5_ includes inorganic ions such as sulfate, nitrate, and ammonia, as well as combustion-form carbon, organic aerosols, metals, and other combustion products. PM_10-2.5_ is dominated by mechanically abraded or ground particles including finely divided minerals such as oxides of aluminum silicate, iron, calcium, and potassium [8].

UFPs are composed of both primary and secondary particulate matters [9]. The primary fraction is the one that is emitted directly from the emission sources and often includes agglomerate/aggregates of smaller particles [9]. Their size is generally in the range between 30 and 100 nm [10, 11]. The primary fraction is generally associated with diesel engines and automobiles and biomass combustion which are thought to initially have been emitted at around the 30 nm diameter size (the so called nucleation mode) and later coagulate into the larger fraction of the ultrafine mode. The secondary fraction is composed of particulate matter formed in the atmosphere and includes sulfuric acid and sulfates and organic reaction products of low volatility [9]. Photoreactions of oxides of nitrogen (NOx) and sulfur dioxide (SO_2_) are involved in this process; both of which are products of combustion. This fraction size is generally in the range between 100 and 200 nm, which is partially distinguishable from other directly anthropogenic sources. 

The role of composition on toxic effects has been explored during the last decade by different authors. The dogma during the 1990's was that the size of the particle was the predominant factor of toxicity, the smaller particles being the more toxic [12]. Nevertheless, during the last 15 years, evidence has linked surface area, reactivity, and different components of the particles with their toxicity [13–15]. The first efforts were done by collecting samples from associated to different sources such as indoor and outdoor [16], different cities [17], or regions within a large city [18] and comparing their *in vitro *toxic effects. In some cases, partial chemical characterizations or determinations of the presence of some components were empirically related with differences in the intensity of toxic effects [19–21]. Later on, comprehensive characterizations were correlated using different statistical models [13–15]. Currently, the characterization of size, physicochemical, and chemical composition is necessary to understand the toxicology of particles. For instance, for nanoparticles (NP), the determination of particle size, the dynamics of agglomeration and aggregation, the area, and the charge are mandatory for any toxicological evaluation [22]. In the field of urban particles, considering that they are complex mixtures, there are no standard measurements of physicochemical and chemical components, but the determination of total carbon, black carbon, transitional metals, nitrates, sulfates, oxidative potential, and polycyclic aromatic hydrocarbons is among the most evaluated components [23, 24]. A recent report of a meta-analysis and multisite time series evaluating elemental carbon, organic carbon matter, sulfate, and nitrate on PM_2.5_ and in its relation to hospital admissions demonstrates that changes in elemental carbon content are associated with cardiovascular hospital admissions [25]. The authors conclude that a stronger communication between risk assessors and epidemiologist would help to better understand the role of the components of air pollutants on population effects.

Among the many components that are present in PM, the biological components seem to play a central role in the biological effects. There is increasing evidence that when PM is inhaled the biological component is responsible for stimulating alveolar macrophages and respiratory epithelial tissue to release proinflammatory cytokines and chemokines. The biological components may also have synergetic effects with other components of the PM, such as diesel exhaust enhancing IgE production and thus facilitating allergic sensitization [26].

These biological components may be released by passive or active mechanisms from plants, soil, biofilms, solid, or liquid sources to become suspended in the air. The measurement of protein associated with PM is considered as a general indicator of how much of the PM comes from a biological source. It represents about 1–4% of the total mass of PM_10_ for urban and rural areas [27, 28].

Airborne biological particles or dust containing biological agents and/or substances of biological origin are important components of the coarse and fine PM. These components are represented by different types of primary or secondary (fragmented biological cells) biological aerosols [29]. The biological matter is predominantly comprised by plant pollen, spores, and microorganisms (mold and bacteria) or microbial metabolites [30, 31] and is related to allergic, toxic, and infectious responses in exposed individuals.

PM may be an efficient carrier of secondary allergens or proinflammatory compounds [32–34]. Recently, good correlation has been found for major allergens, mainly from pollen, and asthmatic patients. In fact, pollen from grasses, weeds and trees, among others were found onto different size of particles [35]. Most of the primary allergens (intact pollen, 10–100 *μ*m) cannot reach the small airways; however, the secondary pollen allergens present in PM_2.5_ can easily penetrate there [36].

Endotoxin lipopolysaccharides (LPSs) are other proinflammatory compounds from microbial origin present in PM. Endotoxin is a component of the cell wall of gram-negative bacteria, and its main source is debris deposited on urban or rural soil. When the LPS is resuspended and inhaled, it stimulates alveolar macrophages and respiratory epithelial tissue to release cytokines/chemokines, initiating an inflammatory cascade [37, 38]. Another biological component with similar effects is the β-1,3-Glucan, a glucose polymer which is structural component of most fungal cell walls. β-1,3-Glucan has been used as an indicator of the presence of mold [39, 40].

## 3. *In Vivo* Studies

**Figure 2:**
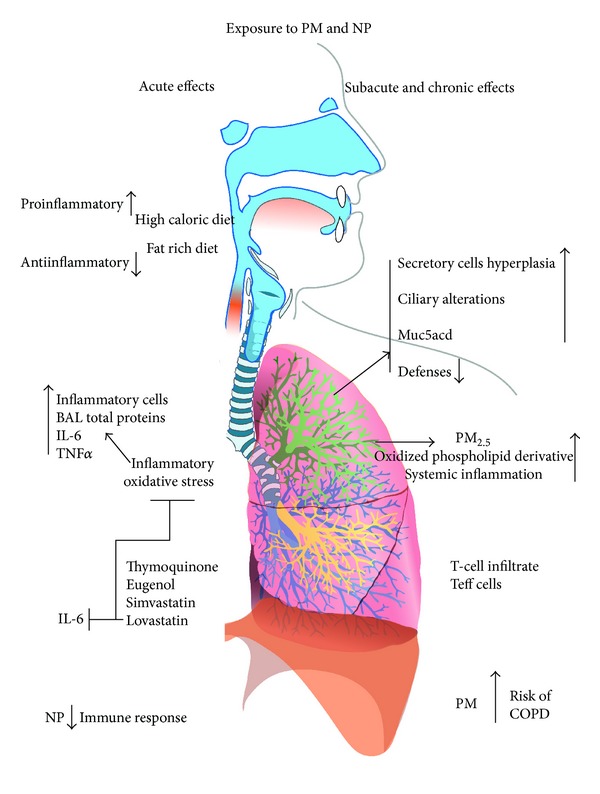
Schematization of the main acute, subacute, and chronic effects induced by inhaled particles and nanoparticles. In the acute side it is interesting to highlight that a high-caloric- and fat-rich diet provides a scenario facilitating proinflammatory effects of particles. Also, Tnymoquinone, eugenol, simstatin, and lovastin have a protective effect *in vivo*. In the subacute and chronic side, it is interesting to highlight the presence of tissue alterations, lung infiltration by T cells, and increases in the risk of COPD.

The main target for inhaled particles is the respiratory system, but there is strong evidence that systemic effects can be induced. We are presenting some of the most relevant studies regarding the local and systemic effects induced by inhaled particles. In Figure 2, we summarize some of the most relevant acute, subacute, and chronic effects induced *in vivo* by particles.

### 3.1. Respiratory Effect of Particles

#### 3.1.1. Acute Effects

Several studies have investigated the respiratory effects of particulate air pollution and nanoparticles. While most of the studies have focused on the respiratory effects following inhalation, intratracheal or intranasal instillation, others have investigated the effects of intravenous, intraperitoneal, or oral administration.

It is well established that pulmonary exposure to particulate air pollution causes inflammation and oxidative stress [41–43]. It has been demonstrated that acute (within 24 h), single-dose intratracheal instillation of diesel exhaust particles (15–30 *μ*g/mouse), a relevant type of PM_2.5_, causes lung inflammation characterized by influx of inflammatory cells, increases total proteins, a marker of epithelial permeability, and oxidative stress [41, 42]. The release of interleukin-6 (IL-6) was found to increase in bronchoalveolar lavage (BAL) fluid at 18 h but not at 4 or 24 h [42]. Similarly, at 18 h time point, airway resistance to methacholine measured invasively using forced oscillation technique increased significantly and dose-dependently following exposure to DEP [42]. Pretreatment with thymoquinone, a constituent of *Nigella sativa*, ameliorated DEP-induced pulmonary effects [42].

Acute exposure (24 h) of healthy mice by intranasal instillation to PM_2.5_ (5 or 15 *μ*g/mouse) collected from the urban area of Sao Paulo caused lung inflammation and oxidative stress and worsened lung impedance in dose-dependent pattern [44]. The same research group has more recently reported that pretreatment of mice with eugenol, a methoxyphenol component of clove oil with anti-inflammatory and antioxidant properties, prevented the changes in lung mechanics, pulmonary inflammation, and alveolar collapse elicited by acute exposure to DEP [45].

The statins are hydroxy-methyl-glutaryl-CoA reductase inhibitors, broadly used in the treatment of hyperlipidemia. They play a key role in the primary and secondary prevention of atherosclerotic heart disease and stroke. Moreover, they have been reported to have potential benefits for a variety of other cardiovascular and noncardiovascular diseases, including cancer, respiratory and neurological disorders [46, 47]. Interestingly, Ferraro and coworkers [48] reported that residual oil fly ash (ROFA) and Urban Air Particle (UAP) from Buenos Aires produced an acute pulmonary injury in mice, characterized by a neutrophilic inflammation, a rise in O_2_^−^ generation, and production of the proinflammatory tumour necrosis alpha (TNF*α*) cytokine. Simvastatin pretreatment had no significant effect per se on any of these biomarkers but prevented the pulmonary cytotoxicity and inflammation induced by ROFA and UAP. More recently, Miyata and coworkers [49] found that pulmonary exposure to PM_10_ in rabbits accelerated the turnover of polymorphonuclear leukocytes (PMNs) by shortening their transit time through the marrow. Interestingly, they found that lovastatin dampens these systemic responses by decreasing the levels of PM_10_-induced circulating mediators (IL-6), thereby suppressing the bone marrow stimulation. The effect of statins was predominant on PMNs in the postmitotic pool as evident by the use of 5′-bromo-2′-deoxyuridine (BrdU/G3). Interestingly, statins also reduced the retention of these newly released PMNs in the lung tissues. These results corroborate the previous finding from the same research group reporting that PM_10_ particles induced systemic inflammatory responses characterized by an increase in systemic proinflammatory mediators such as IL-6 [50]. 

The metabolism of L-arginine plays an important homeostatic role in the airways, through synthesis of the bronchodilating molecule, nitric oxide (NO), from L-arginine, by the nitric oxide synthase (NOS) isozymes. The arginase isozymes (arginases 1 and 2) convert L-arginine into L-ornithine and urea and thus compete with the NOS isozymes for substrate. Arginase overexpression contributes to airways hyperresponsiveness in asthma, and its expression is further augmented in cigarette smoking asthmatics [51]. It has been recently reported that arginase is upregulated following exposure to O_3_ and concentrated ambient particles in murine models of asthma and contributes to the pollution-induced exacerbation of airways responsiveness [52].

The question, whether a diet challenge increases the inflammatory response in the alveolar and the blood compartment in response to carbon nanoparticles (CNP) was investigated by Götz and coworkers [53]. In their study, mice were fed a high-caloric carbohydrate-rich or a fat-rich diet for six weeks and were compared to mice kept on a purified low fat diet, respectively. Bronchoalveolar lavage (BAL) and blood samples were taken 24 h after intratracheal CNP instillation and checked for cellular and molecular markers of inflammation. The authors reported an increase in BAL proinflammatory factors in high-caloric groups and reductions in serum concentrations of anti-inflammatory factors in fat-rich group. They concluded that extended feeding periods, leading to manifest obesity, are necessary to generate an increased susceptibility to particle-induced lung inflammation, although the diet challenge already was efficient in driving proinflammatory systemic events.

Barlow and coworkers [54] assessed the effects of intratracheally instilled PM_10_ collected from London on macrophage clearance in rats *ex vivo*. These authors concluded that acute PM_10_ exposure has an effect on macrophage phagocytosis and chemotaxis that may be deleterious to particle clearance within the alveolar region of the lung. The decrease in chemotactic ability may represent one mechanism that promotes inflammation after increases in ambient PM levels. They also concluded that further investigation is warranted to determine the effects of chronic PM_10_ exposure on macrophage clearance mechanisms. 

NPs induce inflammatory responses and oxidative stress but may also have immune-suppressive effects, impairing macrophage function and altering epithelial barrier functions. The question related to whether exposure to NP may increase the risk of pulmonary infection has been recently investigated [55]. It has been demonstrated that Cu NP exposure impaired host defense against bacterial lung infections and induced a dose-dependent decrease in bacterial clearance [55]. Moreover, it has been demonstrated that acute exposure to DEP by inhalation exacerbates lung inflammation induced by lipopolysaccharide [56].

In an interesting study, the impact of pulmonary exposure to carbon black NP on emphysematous lung injury induced by porcine pancreatic elastase (PPE) was investigated in mice [57]. It has been demonstrated that carbon black NP exacerbates PPE-induced pulmonary inflammation and emphysema. This enhancement may be mediated, at least partly, via the increased local expression of proinflammatory molecules.

TiO_2_ nanoparticles have several industrial applications, and, as such, also have different sizes, shapes, chemistry, and crystalline structures [58, 59]. TiO_2_ occurs in four crystalline polymorphs of which rutile and anatase are most common [60]. Rutile is considered as a more inert form, whereas anatase is an active form of TiO_2_. Anatase and rutile TiO_2_ particles, delivered at similar surface area doses, increased release of lactate dehydrogenase, interleukin-8, and reactive oxygen species, as well as depressed mitochondrial activity in dissimilar patterns in cultured human epithelial cells [61]. *In vivo*, it was observed that ultrafine anatase TiO_2_ particles produced increases in bronchoalveolar lavage inflammatory indicators, cell proliferation, and histopathology compared to ultrafine rutile TiO_2_ particles [62]. However, with both crystalline forms of TiO_2_ particles, pulmonary effects were observed at 24 h and resolved by one week after exposure [62]. Furthermore, it has also been demonstrated that the intratracheal instillation of rutile TiO_2_ nanorods caused upregulation of lung and systemic inflammation and triggered platelet aggregation [63].

TiO_2_-based photocatalysis has attracted extensive interest because of its great advantages in the complete mineralization of organic pollutants in waste water and air [64, 65]. As a popular photocatalyst, TiO_2_ has been widely studied because of its various merits, such as optical-electronic properties, low cost, and chemical stability. Characteristics of TiO_2_ nanoparticles can be modified by several methods to improve their performance. In this context, TiO_2_ nanorods are doped with iron in order to increase their photocatalytic activity [63, 64]. It has been recently demonstrated that exposure to SiO_2_-coated rutile TiO_2_ nanoparticles (cnTiO_2_) caused pulmonary neutrophilia, increased expression of tumor necrosis factor-alpha (TNF*α*) and neutrophil-attracting chemokine CXCL1 in the lung tissue. Uncoated rutile and anatase as well as nanosized SiO_2_ did not induce significant inflammation [66]. More recently, pulmonary exposure to well-characterized rutile Fe-TiO_2_ promotes pulmonary and systemic inflammation and oxidative stress. It also enhances thrombotic potential, heart rate, and systolic blood pressure (SBP) and induces hepatotoxicity. Moreover, rutile Fe-TiO_2_ showed direct toxicity on human lung cancer cells NCI-H460-Luc2 and human hepatoma cells HepG2 [67].

#### 3.1.2. Subacute and Chronic Effects

It has been demonstrated that rats exposed for 6 months to urban air pollution developed secretory cell hyperplasia in the airways and ultrastructural ciliary alterations of the epithelium of the airways, suggesting that chronic exposure to urban levels of air pollution may cause respiratory alterations [68]. Moreover, rats submitted to prolonged exposure to low levels of air pollution experienced deteriorated respiratory defenses against infectious agents [69]. Recently, it has been reported that intranasal instillation of DEP over 60 days increased the expression of Muc5ac in the lungs and the acid mucus content in the nose compared with the 30-day treatment. Moreover, DEP exposure enhanced the total leukocytes in the BAL and the nasal epithelium thickness compared to saline-treated control group [70]. 

Chronic exposure to PM_2.5_ resulted in prominent inflammatory responses in the lung typified by increased levels of oxidized phospholipid derivatives as well as a systemic inflammatory response [71]. In a subsequent study, the same group has extended some of their observations and reported that exposure to PM_2.5_ resulted in increased T-cell infiltration and increased activation of T-effector cells (evidenced by an increase in CD4^+^CD44^+^CD62L^−^ and CXCR3^+^ T cells in the lungs) and suggests a phenotypic switch to a Th1/Th17 phenotype in lung Teff cells. These results have important implications for how PM_2.5_ may detrimentally modulate pulmonary and systemic immune responses [72].

Chronic obstructive pulmonary disease (COPD) is characterized by not fully reversible airflow obstruction that is usually progressive and associated with an abnormal inflammatory response of the lung to noxious particles or gases. The major etiological factor for COPD is chronic oxidative stress as a result of long-term smoking, use of biomass fuels, and air pollution exposure [73]. Lopes and coworkers recently reported a study in which the effects of chronic exposure (2 months) to ambient levels of PM on development of protease (papain) induced emphysema and pulmonary remodeling were investigated in mice [74]. They found that mean linear intercept and the total amount of collagen fibers in parenchyma were significantly greater in the lungs of mice that were treated with papain and exposed to ambient particles compared to those mice treated with papain and exposed to filtered air for 2 months. These increases in destruction of lung parenchyma and in lung collagen content observed only in the group of mice treated with papain and exposed to ambient particles were associated with an increase in the amount of 8-isoprostane expression in lung tissue, suggesting that the increase in oxidative stress is a possible mechanism to explain these alterations [74].

Different types of NPs can cause various inflammatory reactions in the lung. In mice lungs, the toxicity of single-wall carbon nanotube (SWCNT) in causing epithelioid granulomas and interstitial inflammation 7 and 90 days after intratracheal instillation has been shown to be greater as compared with other NPs, like carbon black or quartz particles [75]. However, the significance of the SWCNT-induced inflammation has been a matter of scientific debate. Initially it has been reported that intratracheally instilled SWCNT in rats causes discrete granulomas that were not dose-responsive, and an absence of signs of inflammation in BAL suggested the possibility that large agglomerates of SWCNT caused the granulomas [76]. A second study in rats, using SWCNT aspiration, also reported slight change in the differentials of BAL and a relative lack of histopathologic evidence of inflammation [77]. Studies in mice demonstrated significant inflammation, confirmed that SWCNT-induced inflammation was often granulomatous, and, most importantly, demonstrated that inflammation was present whether the SWCNTs were inhaled or aspirated [75, 78]. It was concluded that SWCNT inhalation was more effective than aspiration in causing inflammatory response, oxidative stress, collagen deposition, and fibrosis as well as mutations of K-ras gene locus in the lung of C57BL/6 mice [79]. Similarly to SWCNT, multiple wall carbon nanotube (MWCNT) exposures by inhalation at concentrations of 5 mg/m^3^ or less for 14 days produced slight evidence of pulmonary inflammation but suppressed T-cell-dependent immune functions [80]. However, the intratracheal instillation of shorter MWCNT failed to show the occurrence of inflammation or fibrosis [81]. Recently, it has been demonstrated that inhalation of MWCNTs for up to 13 weeks caused granulomatous inflammation and pleural thickening at exposure concentrations greater than 6 mg/m^3^. However, influx of inflammatory cells in BAL fluid and interstitial fibrosis wereas demonstrated at exposures above 0.4 mg/m^3^ [82]. 

Pulmonary inflammation caused by NP may also result in changes in membrane permeability, which in turn can result in particle translocation beyond the lung and affecting cardiovascular system [83]. Indeed, it has been shown that NPs have the potential to enter the brain [84] and blood circulation [85, 86] and subsequently other major organs causing inflammation and oxidative stress in these organs. 

### 3.2. Extrapulmonary Effects of Particles

#### 3.2.1. Possible Mechanisms Involved

**Figure 3:**
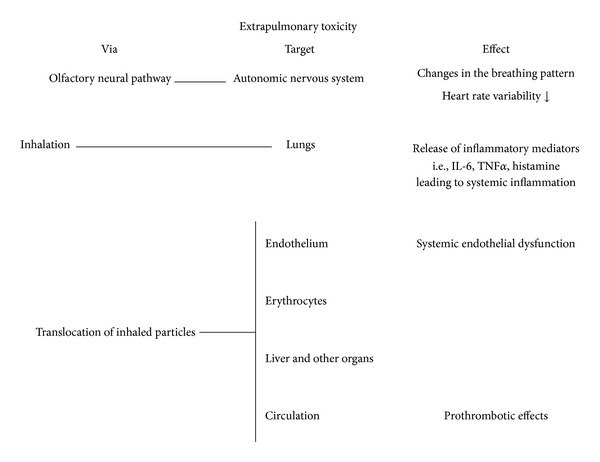
Summary of the main systemic effects associated with particle exposure and the possible mechanisms related to those effects.

Despite the consistency of the epidemiologic observations, the pathophysiological mechanisms linking air pollution to adverse cardiovascular events remain unclear. There are three primary hypotheses that are being investigated to explain the extrapulmonary effect of NP [87, 88], and in Figure 3, we summarize the main mechanisms proposed for the systemic effects. The first one relates the effect of particles to their ability to impact the autonomic nervous system. Studies showed that particulate air pollution exposure is associated with rapid changes in autonomic nervous system balance, favoring sympathetic nervous system activation and parasympathetic withdrawal leading to changes in the pattern of breathing, heart rate, and heart rate variability. Decreased heart rate variability indicates the existence of a state of cardiac autonomy dysfunction and is a risk factor for sudden cardiac death because of arrhythmias [89]. The mechanisms responsible for the increase of the sympathetic drive remain unclear but may involve activations of pulmonary neural reflex arcs and direct effects of pollutants on cardiac ion channels [89]. Inhaled particles may affect the cardiovascular system through inflammatory mediators produced in the lungs and released into the circulation [87, 88]. It has been suggested that inhaled particles may lead to systemic inflammatory response through the release of IL-6, TNF*α* or histamine, and oxidative stress within the lungs and/or systemically [87, 88]. 

Moreover, several studies have shown that nanoparticles, owing to their small size, could avoid normal phagocytic defenses in the respiratory system and gain access to the systemic circulation and therefore to different extrapulmonary sites [85, 86, 90–93]. The UFP can pass from the lungs into the blood circulation in hamsters [86]. Others [91–94] have also reported extrapulmonary translocation of UFPs after intratracheal instillation or inhalation in other animal species. However, the amount of UFPs that translocated into blood and extrapulmonary organs differed amongst these studies. It has also been shown that, following intranasal delivery, polystyrene microparticles (1.1 *μ*m) can translocate to tissues in the systemic compartment [95]. Recent studies [96–98] have provided morphological data illustrating that inhaled particles are transported into the pulmonary capillary space, presumably by transcytosis. Recently, Elder et al. [91] demonstrated that the olfactory neuronal pathway represents a significant exposure route of central nervous system (CNS) tissue to inhaled UFPs. These authors showed that, in rats, which are obligatory nose breathers, translocation of inhaled nanosized particles along neurons is a more efficient pathway to the CNS than via the blood circulation across the blood-brain barrier. They speculated that given that this neuronal translocation pathway was also demonstrated in nonhuman primates, it is likely to be operative in humans as well [84, 91]. In humans, the literature on the translocation of UFPs from the lungs into the blood circulation is still conflicting [85, 99, 100]. However, given the deep penetration of nanoparticulate matter into the alveoli and close apposition of the alveolar wall and capillary network, such particle translocation seems plausible either as a naked particle or after ingestion by alveolar macrophages [98]. Naked particles have been reported to be taken up (and/or adsorbed) by erythrocytes [101] and can presumably be distributed to various organs. The distribution of radiolabelled ultrafine carbon particles, commonly known as “Technegas”, has been investigated after their inhalation by nonsmoking healthy human volunteers [85]. The size of the individualized particles was in the order of 5 to 10 nm, as we confirmed by electron microscopy of the particles. Radioactivity, which was largely particle-bound, as assessed by thin layer chromatography, was detected in blood already after 1 minute, reaching a maximum between 10 and 20 min-, and remaining at this level up to 60 min. Gamma camera images showed substantial radioactivity over the liver and other areas of the body. The presence of radioactivity in the liver is compatible with an accumulation of particles in Kupffer cells, as is known to occur with colloidal particles [102]. More recently, Péry and coworkers [103] developed a physiologically based kinetic model for (99 m) technetium-labelled carbon nanoparticles (Technegas). The model was designed to analyze imaging data obtained from the study of Nemmar and coworkers [85]. It included different translocation rates and kinetics for free technetium and small and large technetium-labelled particles. The authors concluded that the percentage of small particles able to translocate was estimated at 12.7% of total particles, whereas the percentage of unbound technetium was estimated at 6.7% of total technetium [103].

Nurkiewicz and coworkers have studied the effects of inhaled particles and nanoparticles on systemic microvascular endothelium. First, they demonstrated that rats exposed to ROFA or TiO_2_ presented a reduction in their capacity to respond to the Ca^2+^  ionophore A23187, which induce arteriolar dilatation [104]. In other studies, the same group has shown that exposure to ROFA or TiO_2_ NP, by instillation or inhalation, induce systemic microvascular dysfunction [105, 106]. They also found that the nitric oxide (NO) signaling seems to be involved in the endothelial systemic effects of the particles [107].

#### 3.2.2. Acute Effects

Several studies demonstrated that exposure to UFP or DEP caused pulmonary inflammation and prothrombotic events in ear vein of rats or femoral vein and artery of hamsters [108–112]. Mutlu and coworkers [113], showed that exposure to PM triggers IL-6 production by alveolar macrophages, resulting in reduced clotting times, intravascular thrombin formation, and accelerated carotid artery thrombosis [113]. The occurrence of oxidative stress in the DEP-induced acute thrombotic tendency in pial cerebral venules, activation of circulating blood platelets, and lung inflammation have been reported in mice [25]. Moreover, the same authors showed that the antioxidant pretreatment with cysteine prodrug L-2-oxothiazolidine-4-carboxylic acid prevented DEP-induced inflammatory and the resulting thrombotic complications [25]. More recently, the acute (4 and 18 h) effects of DEP on pulmonary and cardiovascular parameters and the protective effect of thymoquinone were investigated in mice [41]. Four h after DEP administration, there were no significant changes in the cells in BAL, lung histology, or pulmonary function test. However, at 18 h after exposure, both lung inflammation and pulmonary function were significantly affected. Conversely, at both 4 h and 18 h, DEP caused systemic inflammation characterized by leukocytosis, increased IL-6 concentration, and reduced SBP. DEP reduced platelets number and aggravated pial arteriole thrombosis. The addition of DEP (0.1–1 *μ*g/mL) to untreated blood induced platelet aggregation. The cardiovascular effects observed at 4 h after DEP exposure did not appear to result from pulmonary inflammation but possibly from the blood translocation of DEP and/or their associated components [41]. However, at 18 h, DEP-induced significant changes in pulmonary and cardiovascular functions and caused lung inflammation. Pretreatment with thymoquinone effectively prevented DEP-induced cardiorespiratory toxicity [41].

It has been reported that TNF*α* is a strong agonist for plasminogen activator inhibitor 1 (PAI-1) expression and has been found to play an important role in PAI-1 regulation in a variety of diseases. In a mouse endotoxemia model, TNF*α* has been found to contribute to the lipopolysaccharide-induced PAI-1 expression [114]. Budinger et al. [115] demonstrated that ambient PM-induced upregulation of PAI-1 disappeared upon treatment of mice with a TNF*α* inhibitor [115]. In line with the later findings, it has been recently demonstrated that repeated exposure to DEP-induced airway inflammation and hyper-reactivity, systemic inflammation, increased SBP, and accelerated coagulation. TNF*α* production was increased both in BAL and plasma. Pretreatment with curcumin significantly inhibited the release of TNF*α* and prevented the respiratory and cardiovascular effects of DEP [116]. 

An important aspect of the epidemiological associations between air pollution and either morbidity or mortality is that the acute adverse effects appear to be most marked in people with preexisting compromised cardiovascular function, such as hypertension or diabetes [89]. To give credibility for these observations, several experimental studies have been designed to test whether and to what extent the effects of particulate air pollution are aggravated, using an animal model of angiotensin II-induced hypertension. Indeed, exposure particulate matter with diameter ≤2.5 *μ*m (PM_2.5_) was found to potentiate angiotensin II-induced hypertension [117, 118]. In addition, PM_2.5_ increased angiotensin II-induced cardiac hypertrophy, collagen deposition, and cardiac and vascular RhoA activation, suggesting that cardiovascular health effects are indeed the results of particulate air pollution exposure [118]. Evidence for exacerbation of thrombotic but not respiratory events was also reported in angiotensin II-induced hypertension in mice [119, 120]. With respect to diabetes mellitus, it has been shown that DEP equally increased airway resistance and caused infiltration of inflammatory cells in the lung of both diabetic and nondiabetic mice. However, the occurrence of oxidative stress, the presence of lung apoptotic cells, and the increase of total proteins, albumin and TNF*α* in BAL fluid were only seen in DEP-exposed diabetic mice suggesting an increased respiratory susceptibility to particulate air pollution [121]. Moreover, the same research group has shown that systemic and coagulation events are aggravated by diabetes in mice acutely exposed to DEP [122]. These authors stated that they may be relevant to the exacerbation of cardiovascular morbidity accompanying particulate air pollution in diabetic patients.

Novel evidence that pulmonary deposition of DEP potentiates the renal, systemic, and pulmonary effects of cisplatin-induced acute renal failure (ARF) has been recently reported by Nemmar and colleagues [123]. These findings highlight the importance of environmental factors such as particulate air pollution in aggravating ARF.

Several studies have showed that nanoparticles, owing to their small size, could avoid normal phagocytic defenses in the respiratory system and gain access to the systemic circulation and therefore to different extrapulmonary sites [83, 84, 90–93, 103, 123]. To specifically determine the effect of translocated particles, it has been recently demonstrated in both normotensive and spontaneously hypertensive rats that 24 h following their systemic administration, DEP affected blood pressure and caused pulmonary inflammation assessed by BAL [124, 125]. In a subsequent study in rats, it has been reported that i.v. administration of DEP (0.02 mg/kg) caused acute systemic effects mainly at 6 h and 18 h but not at 48 h or 168 h following particle exposure. While DEPs were found in lungs, heart, liver and kidneys, the histopathological changes were only seen in the lung. This implies that, at the dose and time-points investigated, DEP can cause inflammation in the lungs but not in other organs, suggesting that pulmonary tissue is the predominant site for inflammation based on the mode of delivery of DEP in this study [126]. Furthermore, it has been shown that ultrafine TiO_2_ induces acute lung inflammation after i.p. administration and exhibits additive or synergistic effects with LPS, at least partly, via activation of oxidant-dependent inflammatory signaling and the NF-kappaB pathway, leading to increased production of proinflammatory mediators [127]. Geys et al. [128] have investigated the toxicity of quantum dots which have numerous possible applications for *in vivo* imaging. QDs caused marked vascular thrombosis in the pulmonary circulation, especially with carboxyl QDs. QDs were mainly found in lung, liver, and blood. Thrombotic complications were abolished, and P-selectin was not affected by pretreatment of the animals with heparin. 

#### 3.2.3. Subacute and Chronic Effect

Akinaga and coworkers [129] reported a study in which mice were continuously exposed, since birth, in two open-top chambers (filtered and nonfiltered for airborne particles ≤0.3 *μ*m) placed 20 m from a street with heavy traffic in downtown Sao Paulo, 24 h per day for 4 months. They found that air pollution induced mild but significant vascular structural alterations in normal individuals, presented as coronary arteriolar fibrosis and elastosis.

PM has been shown to cause significant decreasing patterns of heart rate, body temperature, and physical activity in mice lacking apolipoprotein (ApoE^−/−^) over 5 months of exposure to concentrated ambient PM, with smaller and nonsignificant change for C57 mice [130]. 

Sun and coworkers demonstrated that ApoE^−/−^ mice exposed to concentrated regional northeastern PM_2.5_ for 6 months (6 h/day for 5 day/week) in conjunction with high-fat chow potentiated plaque development markedly increased vascular inflammation (CD68^+^ macrophage infiltration and inflammatory nitric oxide synthase (iNOS) expression) and vessel wall markers of oxidative stress [131]. Plaque progression was accompanied by alterations in vasomotor tone, including decreased endothelial-dependent vasodilatory function and heightened vasoconstriction to adrenergic stimuli. The same research group confirmed their findings by another set of experiments which was performed using an identical protocol of exposure but involving an apoE^−/−^ model and a double-knockout (DK) model of ApoE^−/−^ and low-density lipoprotein (LDL) receptor deficient mice (DK mice) to concentrated ambient PM_2.5_ for 6 h a day for 5 days/week for up to 5 months. Although quantitative measurements showed that PM_2.5_ exposure increased atherosclerotic lesions in the apoE^−/−^ mice, changes produced by PM_2.5_ in DK mice were not statistically significant [132]. In subsequent set of experiments, it has been shown that chronic ambient exposure to PM_2.5_ increased tissue factor expression in macrophages and smooth muscle cells in atherosclerosis [133]. They also reported specific recruitment of monocytes into microcirculatory tissue niches (i.e., adipocytes) in response to long-term PM_2.5_ exposure [134]. These experiments suggest a key role for PM_2.5_ in the activation and mobilization of innate immune cell populations. 

Long-term cardiovascular effects of inhaled nickel hydroxide NPs (nano-NH) in hyperlipidemic, ApoE^−/−^ mice were investigated by Kang and coworkers [135]. Mice were exposed to nano-NH at either 0 or 79 *μ*g Ni/m^3^, via a whole-body inhalation system, for 5 h/day, 5 days/week, for either 1 week or 5 months. Inhaled nano-NH induced significant oxidative stress and inflammation in the pulmonary and extrapulmonary organs, indicated by upregulated mRNA levels of antioxidant enzyme and inflammatory cytokine genes; increased mitochondrial DNA damage in the aorta; significant signs of inflammation in BAL fluid; changes in lung histopathology; and induction of acute-phase response. In addition, after 5-month exposures, nano-NH exacerbated the progression of atherosclerosis in ApoE^−/−^ mice [135].

Emmerechts and coworkers have investigated how continuous traffic-related air pollution exposure affects haemostasis parameters in young and old mice. Young (10 weeks) and old (20 months) mice were placed in an urban roadside tunnel or in a clean environment for 25 or 26 days. They found in old mice that subchronic exposure to polluted air raised platelet numbers, von Willebrand factor, soluble P-selectin, and microvesicles, collectively substantiating a further elevation of thrombogenicity, already high at old age [136].

There is a potential for neurodegenerative consequence of particle entry to the brain. Histological evidence of neurodegeneration has been reported in both canine and human brains exposed to high ambient PM levels, suggesting the potential for neurotoxic consequences of PM entry [137, 138]. PM-mediated damage may be caused by the oxidative stress pathway which can enhance the susceptibility for neurodegenerative diseases. The relationship between PM exposure and central nervous system degeneration can also be detected under controlled experimental conditions [137, 138]. Morphometric analysis of the central nervous system of ApoE^−/−^ mice exposed to concentrated ambient air pollution showed that the brain is a critical target for particulate air pollution exposure and implicated oxidative stress as affecting factor that links PM exposure and susceptibility to neurodegeneration [137, 138]. Further experimental studies are needed to clarify the effect and mechanisms underlying the neurotoxicity of particulate air pollution.

## 4. *In Vitro* Studies

*In vivo* models give a good insight of the toxic effects of particles, and considering the multiple interactions of different cell types in the lung, the complex responses are well documented, but the cellular mechanisms related to the specific responses become very difficult to clarify. In this regard, the *in vitro* models are used as a main tool to evaluate the cellular mechanisms related to the exposure to particles.

There are several approaches to evaluate the toxic effects of particles on cells that have been suggested or pointed as targets of PM and NP. Single cell cultures, cocultures, multiple cocultures, exposure under submerged conditions, and exposure under air-liquid interface are among the main strategies. We are discussing some of the most significant advances on the evaluation of PM *in vitro* toxicology. In Table 2, we summarize the most relevant *in vitro* evidence supporting the observed *in vivo* effects.

### 4.1. Particle Properties Linked to Primary Cell Interaction

In the lung, the particles may interact with the lung lining fluid and the epithelial cells. In addition the particles may be taken up by macrophages and other immune cells by phagocytosis or pinocytosis. The interaction of particles with the cellular plasma membrane and its receptors and ion channels may directly trigger a biological response. The important DEP-induced reactions often start from constituents leaking from the particles including metals and various PAHs, including derivatives like nitro-PAHs and various oxo-PAH (quinones). The relative position of such components on the particle is most likely of importance since just adding back extracted components may result in less effects than the native particle exerts [139]. Furthermore, the combination of particle constituents like endotoxins and chemicals in organic fraction may elicit more than additive cytokine response effects [15]. On the other hand, with regard to genotoxic effect, the response will be higher in the extracts as more of the carcinogenic PAH will be available to the cells [140, 141].

Although particle uptake in epithelial cells has also been reported to occur [142], most biological responses triggered by particles in these cells do not seem to depend on particle uptake [143]. Particles as such have been reported to trigger biological effects via acellular reactive oxygen species (ROS) formation. However, DEP-induced immune responses in A549 cells were reported to depend on activation of cellular ROS-formation via the NADPH oxidase [144]. Furthermore, emerging evidence suggests that particle constituents are able to bind to or otherwise activate various membrane and cytosolic receptors. Obviously, both AhR-ligand binding as well as reactive electrophilic PAH metabolites covalently binding to DNA are caused by chemical constituents released from the particle [143, 145]. 

### 4.2. *In Vitro *Studies with Implications to Various PM-Induced Cardiovascular Effects and Various Lung Diseases Including Cancer

As we have seen in previous sections, damage of the lung epithelial lining may have important implications with regard to pathogen diseases, asthma, and allergy. Direct or indirect induced chronic inflammation is also considered to be central element in various cardiovascular diseases, COPD, and a likely part of cancer development.

Regarding the latter, there is growing evidence suggesting that air pollution exposure increases risk of lung cancer [146, 147]. The components generally considered being of most interest for such effects are particles in the ultrafine (PM_0.1_) and fine fraction (PM_2.5_) including DEP and wood smoke particles (WSP) [148]. However, more recent *in vitro *evidence indicates that also the larger PM_10_ particles might play a role in cancer development through mechanisms such as damage to the lung epithelial cells, disruption of tight junction and gap junction, effects of cell proliferation including cytotoxicity, release of inflammatory mediators like chemokines and cytokines, changes in gene expression via receptor binding, and various forms of cellular DNA damage, including epigenetic changes. It is also possible to study *in vitro* effects of particle exposure on the later stages of cancer development like chromosomal instability and cell migration, which are important parts of tumor promotion and metastasis. However, we are not aware of that such studies have been published.

#### 4.2.1. Tight Junction

Tight junctions between the epithelial cells represent an important barrier for the body protecting the rest of tissue and organs from exposure to various pathogenic intruders like virus, bacteria, fungi, air pollution PM, and various particle-bound allergens. Exposure to such components can result in infections and allergic/asthmatic reactions. If combined with PM exposure, the end result may be more chronic inflammatory reactions, which is considered to be an important part of many pulmonary diseases including COPD and cancer development. Geys and coworkers showed that the transepithelial electrical resistance (TEER) is linked to the tight junctions and the correlation between the TEER value and the translocation of particles through cellular monolayers [149]. Using an *in vitro* triple cell culture model consisting of human epithelial cells (16HBE14), monocyte-derived macrophages and dendritic cells, it was recently demonstrated that macrophages, and dendritic cells create a transepithelial network between epithelial cells to capture antigens without disrupting the epithelial tightness [150]. Using a similar model, Lehmann and coworkers [151] observed that a high concentration of DEP (NIST 2975, 125 *μ*g/mL) can modulate the tight junction occluding mRNA in the cells of the epithelial defense system. In this connection, it is also interesting to note that NIST 2975 DEPs have been reported to increase the release of metalloproteinase MMP-1 from human lung epithelial cells (A549 and NCI-H292). MMP-1 is involved in the degradation of collagen and can thus damage the lung epithelial barrier [144]. These findings suggest that DEP can contribute to structural changes in the epithelial lining with inflammatory and possible carcinogenic implications.

#### 4.2.2. Gap Junction Intercellular Communication (GJIC)

GJIC is one way of intercellular exchange of low molecular weight molecules between adjacent cells. Chemically induced alterations in this type of communication have been found to result in abnormal cell growth and behavior and is considered to be an interesting assay for *in vitro* studies of chemicals that may act as tumor promoters [152]. Bay/bay-like regions of PAH have been reported to be potent inhibitors of GJIC [153]. Interestingly several high molecular weight PAHs with known strong carcinogenic properties possessed only weak (dibenzopyrenes) or no inhibition potency (dibenzofluoranthenes, naphtho[2,3-a]pyrene, and benzo[a]perylene) [154]. Furthermore, the PAH-induced inhibition of GJIC occurs in the absence of PAH metabolism and aryl hydrocarbon receptor (AhR) binding [155]. More probably, unmetabolized PAH changes GJIC through direct interaction with unknown factor(s) in the cellular membrane. In line with this, DEP has been reported to inhibit GJIC [156–158]. The GJIC-effects of a fractionated DEP extract were found to be due to components in the polar fraction, while the less polar nitro-PAH fraction showed the strongest mutagenic potential (Ames test) [158].

#### 4.2.3. Cell Proliferation and Cytotoxicity

Measuring cellular proliferation and cytotoxicity has been used as one of the primary toxicity tests for particulate matter [15, 16, 159]. With relatively simple methods, differences in the intensity of cytotoxicity have been demonstrated. Equal masses of urban PM collected in different cities, or within a large city, associated with different sources presented differences in cellular proliferation and cytotoxicity [16, 17]. These results were of main interest to evaluate the role of different components of the toxic effects of particles and therefore identifying components such as endotoxin, organic carbon, and some elements, as the components associated to the cytotoxicity [14, 16, 17].

Increased cytotoxicity is often followed by proliferative stimuli considered to be of great importance for both fixation of the primarily DNA lesion as well as for tumor promotion phase. A number of compounds in DEP are cytotoxic; other compounds are known to be DNA damaged thus resulting in G1-arrest and/or accumulation in S-phase due to reduced DNA synthesis [160, 161]. However, DEPs also include compounds which may affect cell proliferation in other ways. Two nitrophenols isolated from DEP 3-methyl-4-nitrophenol (4-nitro-m-cresol, PNMC) and 4-nitro-3-phenylphenol (PNMPP) have been reported to have estrogenic and antiandrogenic activities. Most interestingly, proliferation of MCF-7 cells was stimulated by PNMC, PNMPP, and estradiol-17beta and the antiestrogens 4-hydroxytamoxifen and ICI 182,780 inhibited the proliferation [162]. Crude extract of DEP exhibited both estrogenic and antiestrogenic activities. Estrogenic activity of crude extract and some fractions was induced through estrogen-receptor- (ER-) mediated pathways. In particular, the acid polar fraction of DEPs, which contains phenols, induced high levels of estrogenic activity compared to other fractions [163]. 

An important part of the known carcinogens found on air pollution particles is various PAHs. Some of these have also been reported to have mitogenic potency. More specifically, weak mitogenic effects have been reported, suggested to occur via increased Ca^2+^, activation of epidermal growth factor receptor (EGFR) and insulin receptor [164–167]. Furthermore, disruptions of contact inhibition via AhR-dependent induction of JUN-D/cyclin [168] have been observed. This type of effect obviously would also result in increased cell proliferation. Most interestingly, it is known for a while that several of these have so-called “stealth properties” [169–171]. This is a property by which reactive metabolites are able to covalently bind to the DNA without easily being detected by the cells defense system. More specifically, some reactive PAH metabolites bind to DNA without triggering a G1-arrest. An increase in p53 seems to be induced but not a p21waf1/cip1-inhibition of p53 transcriptional activity. Furthermore, some PAH seems to induce mdm2 which may reduce the p53 activation [172, 173]. AhR-dependent inhibition of E2F1-dependent apoptosis [174] reduced p53 nuclear translocation, stimulation of cell survival signals, and inhibition of DNA damage induced apoptosis have been reported after exposure to certain PAH [175, 176]. Most importantly, such chemicals would change the balance between cell death and cell survival and cell proliferation following a DNA damaging event. If not compensated with increased DNA repair, the end result would necessarily be increased formation of mutation. Furthermore, reactive metabolites that react to a larger degree with DNA than other macromolecules in the cells will have a higher mutagenic potential [177, 178]. In line with this, it has recently been reported that several environmental pollutants including the carcinogenic PAH benzo[a]pyrene may change plasma membrane characteristics, thereby altering cell physiology and the balance between life or death of a cell [179].

#### 4.2.4. Inflammatory Mediators

Several cytokines have been found to function as proliferation and/or survival factors, for example, IL-6, IL-8, and IL-1β [180] and which may have implications for several lung diseases including cancer development. Thus, a number of studies *in vitro* have elucidated the inflammatory potential of various air pollution particles [181]. In studies with BEAS-2B bronchial epithelial lung cells DEP from a pre year 2000 engine increased the release of chemokines such as IL-8 [182]; whereas EURO-4 DEP-induced typically IL-6 and IL-8, but also to a certain degree CCL5, CXCL10, and IL1β [139]. Increased CCL5 (RANTES) after DEP exposure (pre year 2000 engine) has also been reported by Hashimoto et al. [183]. Often the induced expression and release of pro-IL-1β found to be due to a combination of endotoxins and other particle components [184]. In general, oxidative stress is considered an important mechanism of particle-induced toxicity and inflammation [181] in addition to other pathways of particle effects. Direct ROS-formation by DEP may arise from enzymatic metabolism of organic compounds such as PAHs or directly [185, 186]. Possible mechanisms also include a direct activation of the membrane bound NADPH oxidase enzyme, inducing the formation of ROS near the plasma membrane [187]. A correlation between NADPH oxidase activation and proinflammatory response has been reported using both *in vitro* and *in vivo* systems exposed to air particles [188]. As seen typically in studies of air pollution collected from cities, there seems to be large seasonal differences in PM_10_ and PM_2.5_ both with regard to chemical composition and their biological effects as measured as proinflammatory cytokine release and cytotoxicity [184]. The summer PM_10_ exhibited a higher proinflammatory potential, partly due to biological components such as LPS as also previously reported by others [189, 190]. Typically induced cytokines reported include IL-6, IL-8, and IL-1β. However, it should be emphasized that no simple mechanism exists that explains all cellular effects, and in some cases contradictory results have been observed for IL-6 and IL-8 [191]. Furthermore, oxidative stress alone seems to be insufficient to induce proinflammatory responses in lung cells, pointing also to other mechanisms [192, 193]. Moreover, the mechanisms of particle-induced toxicity are likely to change with increasing concentrations.

Of particular interest, recent studies show that DEP may induce Ca^2+^ influx through proteinase-activated receptor-2 (PAR-2) mediated activation of TRPV4 channels in human bronchial epithelial cells. This effect is probably linked to IL-8 responses in bronchial epithelial cells induced by multiple compounds found in ambient air [194]. Studies also suggest that DEP exposure activates EGFR signaling [194]. The activation of EGFR signaling through cleavage and release of membrane bound transforming growth factor (TGF-*α*) by the metalloproteinase TNF*α* converting enzyme (TACE or ADAM17) seems to be a universal mechanisms of IL-8 regulation in airway epithelial cells by multiple endogenous and exogenous compounds, including DEP and various air pollution components [194]. It is also possible the particle/DEP-linked formation of reactive metabolites more directly could interfere with various cell signaling pathways or effect organelles, thereby initiating inflammatory reactions. 

The vascular endothelium plays a central role in the inflammatory process and cytokine production, and various cellular signaling pathways trigger this response. Considering the evidence that particulate matter can translocate from the lungs within few minutes after exposure [85], the inflammatory signal could reach the vascular endothelium by direct exposure to particles. In this regard, several studies have shown that PM and NP induce endothelial dysfunction after exposure [16, 195–198]. The expression of early (E-Selectin, P-Selectin) and late adhesion molecules (ICAM-1, VCAM-1, PECAM-1) was associated with the presence of endotoxin [199], the size of the particles [200], and the oxidative stress induced by the particles and nanoparticles [201, 202]. Despite the evidence provided by these studies, there is no certainty of the amount of particles that can translocate, and therefore, the experimental conditions of exposure are always of concern.

*In vivo*, the epithelial cells or macrophages, or any cell that is interacting with a particle, have an interaction with other cell types, and those interactions may exacerbate or inhibit the inflammatory response. Therefore, the single cell cultures have the limitation of not evaluating those interactions. Cocultures of two or more cell types may help to improve the *in vitro* studies. A study using multiple cell cocultures of human lung epithelial cells, macrophages, mast cells, and endothelial cells demonstrated that when cocultures were exposed, a stronger cytokine production was observed in comparison to the responses obtained on single culture [203]. These types of models help to evaluate if the first contact of PM or NP with relevant cells is enough to induce an endothelial activation that may lead to systemic effects. In this regard, a modification of the model described by Alfaro-Moreno et al., using a coculture where the endothelial cells and epithelial cells are seeded on both sides of a membrane, demonstrated that by exposing the epithelial cells, an activation of the endothelial cells was evident within 24 h of exposure [204].

It is interesting to note that inflammatory diseases like asthma and COPD have been suggested to confer an increased risk of lung cancer, although this implication may not be straightforward [199, 200]. The hypothesis is based on that the release of inflammatory mediators (chemokines and cytokines) like IL-1β directly or via increased cytotoxicity (release of DAMP molecules) may result in an increased number of neutrophils/macrophages in the lung. Thus, several *in vivo* studies on other chemicals have reported that the recruitment of such cells will result in increased release of ROS molecules that might exacerbate the increased toxicity and thereby amplify the inflammatory process. Augmented inflammation in a tissue will increase the oxidative/nitrosative stress and lipid peroxidation (LPO), thereby further generating excess ROS, reactive nitrogen species, and DNA-reactive aldehydes. Miscoding etheno- and propano-modified DNA bases are generated interalia by reaction of DNA with these major LPO products [201]. The resulting highly cytotoxic environment will also create surroundings that favor selection of cells with mutations in p53, making them more resistant to cell death [202]. Additional putative mechanisms include impaired or imbalanced DNA repair pathways. In this way, persistent oxidative/nitrosative stress and excess LPO are induced by inflammatory processes in a self-perpetuating process and cause progressive accumulation of DNA damage in target organs including the lung [201].

However, the role of particle-induced inflammation in lung cancer development is very complex. During the latest years, it is becoming increasingly clear that cytokines and chemokines can have a profound role in not only progression, but also rejection of tumors [205].

#### 4.2.5. Changes in Gene Expression via Receptor Binding

Certain changes in phenotypes might give increased probability to development into cancer cells. Regarding exposure to urban air particles, it is well known that some of these like DEP and wood initiate various AhR responses [145, 161, 206]. This is explained by the fact that potent AhR ligands such as PAH and dioxins are released from the particles. The activation of the AhR results in increased metabolism of xenobiotics, and changes in the balance between several metabolic and detoxification pathways are often seen [177]. These types of changes may have important implications for the cells, as more or less reactive metabolites are central for cancer initiation, promotion and for inflammatory reactions. Furthermore, this receptor has also very important physiological functions that extend beyond specific metabolism of xenobiotic, including effects on proliferation, contact inhibition and migration, and immune regulation [145]. All these process may have important implication for cancer development.

#### 4.2.6. Epigenetic Changes

Gene transcription is activated when specific CpG sites are demethylated and histones are acetylated, and, conversely, silenced when sites are methylated and histones deacetylated. Furthermore, in addition to oncogenes, tumor suppressors and miRNAs are the major regulators of signaling in the cancer phenotype [207, 208]. Thus, possible implications of air pollution particle-induced epigenetic changes should clearly be explored *in vitro* systems as these endpoints may become important biological markers for epidemiological studies in the future. 

#### 4.2.7. Genotoxicity

It is well documented that different types of particles, their extracts as well as single components attached have genotoxic effects in human and animal studies *in vivo* [209] as well as *in vitro.* After exposure of cells in culture to different types of PM, several studies have shown that cells may be arrested in various parts of the cell cycle [160, 161, 210, 211]. Most often, such effects have been linked to DNA damage. Various forms of DNA damage have been reported after exposure to PM. The DNA damage includes DNA single-strand breaks, alkali-labile single-strand DNA breaks, and various forms of oxidative DNA damage including oxidized guanines measured as 8-oxo-7,8-dihydroguanine (8-oxoGua) and lesions detected as formamidopyrimidine DNA glycosylase (fpg) sites by the comet assay [161, 205]. Often this type of damage is associated with the formation of micronuclei and chromosomal damage. In line with this, positive effects of DEP on chromosome aberration and sister chromatid exchange have been reported in V79 cells without any additional activation system added [212]. The organic extract of PM_2.5_ was reported to generate DNA breakage and micronucleus formation using BEAS-2B cells as a model system. Testing of various fractions in comet assay with fpg in this system suggested a possible role of ROS and that aliphatic/chlorinated hydrocarbons, PAH/alkylderivatives, and nitro-PAH/ketones/quinones may be important causative agents of the genotoxic effects [213]. Furthermore, it should be noted that DEP-extractable organic matter (EOM) has been reported to have a substantial higher capacity than the individual classic carcinogenic PAH with regard to induce oxidative damage to DNA in HepG2 cells [214].

While many reports focus on DNA breaks and/or oxidative-DNA damage with regard to cancer development [209], others link the PM-induced genotoxic and carcinogenic effect to the “classic” carcinogenic PAH giving rise to DNA adducts, often analysed by the ^32^P postlabelling study [214]. Such PAH needs to be metabolically activated to reactive, electrophilic metabolites that covalently bind to DNA. Both acellular as well as various cells *in vitro *are used. The adduct levels formed are linked to PAH levels in extracts, fractionated extracts, or single PAH compound tested separately [214]. The results from such studies indicate that most DNA adducts detected in cells incubated with extractable organic matter (EOM) from ambient air have their origin in the low concentrations of carcinogenic PAH representing a very low part of EOM total mass (0.03–0.17%; [199]). In general, the bulky DNA adducts are more often associated with high potency to form gene mutations, considered to be of particular important for the initiation phase of cancer development. 

An important point in evaluating genotoxic potential is the use of a metabolic activation system with sufficient ability and capacity to activate these carcinogenic PAHs. Certain types of lung epithelial cells (e.g., Clara and type II) *in vivo* have a relatively high level of CYP enzymes due to exposure to AhR ligands (various PAH, dioxins) linked to ambient air particles. Accordingly, several publications have shown DNA adducts, DNA breaks and oxidative DNA damage(s) after exposure to ambient particles [215–218]. Thus, lung epithelial cells will in the *in vivo* situation have a clear capacity to activate various carcinogenic compounds including PAH. However, in contrast, the various lung epithelial cell lines as well as primary lung cells from laboratory animals used *in vitro* have a much lower capacity to activate such compounds. Such cells are thus, not always, the best choice to use when testing for genotoxic effects of various ambient air particle types. Interestingly, some liver-derived cell lines seem to have a more interesting capacity to metabolic activated PAH somewhat more similar to human *in vivo* situation; although metabolic enzyme profile in liver will be different compared to lung. Such models have nevertheless been suggested to represent better *in vitro* models for investigating the genotoxic potential of complex mixtures containing PAH [214, 219]. Another important aspect is to use a test system that can detect the primary DNA damage of importance. This could include technique such as the ^32^P postlabeling technique to detect the larger and bulky DNA adducts. In order to detect and evaluate DNA damaging constituents which causes smaller DNA adduct/and other DNA lesions, the comet assay with or without addition of fpg is a good supplement [220].

Although not presently in use, it is possible to test the capacity of particles and their extracts to transform epithelial cells *in vitro*, representing a test of both “initiating” as well as tumor “promoting” properties. In a transformation assay using BALB/c 3T3 cells, DEPs have reported to cause morphological transformation [212]. Similarly, it was reported that DEP and two related compounds, 1-nitropyrene (1-NP) and dibenzo(a,i)pyrene (DBP), are capable of transforming rat tracheal epithelial cells [221]. Various coculture systems also add important information to the problem of a “relevant metabolic activation model” when testing genotoxic effects of PM *in vitro*. In a recent study, results supporting the notion that highly reactive benzo[a]pyrene (B[a]P) derived metabolites produced within human alveolar macrophage could be transferred to a secondary target epithelial cell line were presented [222]. Such findings have in addition important *in vivo* implications when explaining possible mechanisms involved in ambient air induced lung cancer. By using DNA repair capacity *in vitro* many important aspects of the role of DNA repair in maintaining genetic stability and preventing carcinogenesis can be elucidated [223]. Furthermore, studies and analyses of polymorphisms of DNA repair genes involved in nucleotide excision repair (NER) may turn out to be useful biomarkers to identify individuals susceptible to DNA damage resulting from ambient air exposure [224]. Also the level of proteins involved in the DNA response like gamma-H2AX, p53, and p21 (WAF1) protein levels has been analyzed and linked to PM-induced genotoxic and cytotoxic effects [161, 225]. Most interestingly, it has been reported that ambient air PM greatly inhibits nucleotide excision repair (NER) for ultraviolet (UV) light and benzo[a]pyrene diol epoxide (BPDE) induced DNA damage in human lung cells. PM increased both spontaneous and UV-induced mutagenesis, suggesting that the carcinogenicity of PM may act through its combined effect on suppression of DNA repair and enhancement of DNA replication errors [226]. 

## 5. Conclusions

Urban air pollution consists of an extremely complex mixture of gaseous and particulate agents. The majority of published studies concur to the statement that whilst gaseous pollutants, such as ozone or SO_2_, play a significant role, the unifying element of the adverse health effects of urban air pollution consists of respirable PM [1, 88]. Many studies using animal models have been performed to elucidate PM effects in different organs, in relation to different diseases. With respect to acute effects, most studies have focused on inflammatory responses, and relatively few studies have included more disease-specific responses, perhaps with the exception of studies on allergy-related responses. In contrast more studies on chronic effects have elucidated disease-related processes, such as DNA damage, lung parenchyma destruction, increased plaque volume in arteries, lung fibrosis, or granuloma formation. An increased focus on more direct disease-related parameters in models that closely resemble the human disease pattern would improve the usefulness of the *in vivo* models. 

Since the *in vitro* models prove themselves to be most useful to study mechanistic responses, such as initiation events of inflammatory effects or genotoxicity, it would be of interest for the interpretation of results if the *in vivo* studies could also to a greater extent cover mechanistic effects, to discover a possible coherence of results with the *in vitro* studies. Whereas the relationship between some *in vitro* end points, particularly with respect to genotoxicity and indicators of cancer development and disease, has been established; with respect to other end points, this relationship has not been fully developed. Improved *in vitro* models that seek to cover this field need to be further developed.

The *in vitro* models have proven useful in studying the importance of a range of particle sizes and components. For example, evidence suggests that the ultrafine fraction of these particles shows more toxicity at equal mass concentrations compared to larger particles, because of their increased reactivity, surface area, and particle number on a mass basis. Furthermore, a coherence of certain *in vitro* cellular effects and responses in biopsies from human volunteers has been shown for the exposure to diesel exhaust particles [194, 227]. On the other hand, sometimes very high concentrations used in *in vitro* models suggest caution in the interpretation of *in vitro* results and again points to the development of more sensitive models. 

Nanotechnology develops products with highly different physical and chemical properties, and they are also used in a variety of areas such as diagnosis, imaging, drug delivery, information, and communication technologies, and their extensive use in the consumer and industrial products is just beginning to emerge [87]. Thus, in order to cope with such a variation of type of material and use, structure activity and *in vitro* studies will be of help [87].

The increased risk of respiratory and cardiovascular diseases requires additional toxicological studies to be performed and specific measures to be taken for environmental PM and newly developed engineered NP.

## Figures and Tables

**Table 1:** Comparison of the WHO guidelines and standards from different countries. Modified from WHO air quality guidelines, global update, 2005, a report on a Working Group meeting, Bonn, Germany, 18–20 October 2005 [5].

Selected air quality guidelines and standards				
Source	PM_10_ (*μ*g/m^3^)		PM_2.5_ (*μ*g/m^3^)	
	1 year	24 hours	1 year	24 hours
WHO [2]	20	50	10	25
European Union	40	50	25	
United States	50	150	12	35
California	20	50	15	65
Japan		100	12	65
Brazil	50	150		
Mexico	50	120	15	65
South Africa	60	180	15	65
India (sensitive populations/ residential/industrial)	50/60/120			
China (Classes I/II/III)	40/100/150	50/150/250		35

**Table 2:** *In vitro* evidence that supports and provides plausible mechanisms for the *in vivo* observed effects induced by PM and NP.

*In vitro* evidence supporting the observed* in vivo *effects	
*In vivo* observed effect	*In vitro* evidence
Oxidative stress	ROS increases via NADPH-oxidase in lung epithelial cell exposed to PM.
Local and systemic inflammation	Secretion of IL-1b, IL-6, IL-8, TNFa, MCP-1, and so forth, by lung cells, macrophages, and cocultures.
Hyperplasia	Proliferative stimuli induced by extracts of DEP components.
COPD	Increased cytotoxicity on exposed cell cultures.
Systemic and endothelial dysfunction	Endothelial cell activation by direct contact with particles or indirectly induced in cocultures where pneumocytes, macrophages, and other cell are exposed.
Particle translocation	Changes in the TEER values related to tight junctions Macrophage-dendritic transepithelial cells network alterations in the GJIC.
